# Intraspinal leiomyoma: A case report and literature review

**DOI:** 10.3892/ol.2014.2299

**Published:** 2014-06-30

**Authors:** TAO YANG, LIANG WU, XIAOFENG DENG, CHENLONG YANG, LEI ZHAO, YULUN XU

**Affiliations:** Department of Neurosurgery, Beijing Tiantan Hospital, Capital Medical University, Beijing 100050, P.R. China

**Keywords:** leiomyoma, spinal

## Abstract

Leiomyomas are benign tumors which are predominantly found in the genitourinary and gastrointestinal tracts. Leiomyomas in the spine are extremely rare. The current study presents a case of a 35-year-old female with intraspinal leiomyoma who presented with low back pain and weakness in the left leg of two months. Computerized tomography and magnetic resonance imaging revealed an epidural mass at the T11–12 levels. The patient underwent a T11–12 laminectomy through posterior approach, achieving total removal of the tumor with a well-demarcated dissection plane. Pathological examination demonstrated a leiomyoma. Postoperatively, the patient showed a significant improvement in neurological function. Although intraspinal leiomyoma is extremely rare, it should be considered in the differential diagnosis of spinal lesions in females. The diagnosis is predominantly dependent on a pathological examination. Gross total resection is recommended for its treatment, however the prognosis remains poor. Post-operative hormonal therapy may be useful in controlling tumor recurrence.

## Introduction

Leiomyomas are benign tumors composed of smooth muscle and vascular collagenous tissue mainly occurring in the uterus (1). Leiomyomas account for <2% of soft tissue tumors, with an incidence rate of 8 per million (2). Leiomyomas in the spine are extremely rare, with only eight reported cases of intraspinal leiomyoma (1–8). Epstein-Barr virus, the human immunodeficiency virus, and immunosuppression may be cofactors in the pathogenesis (9–11). Leiomyomas usually cause spinal cord compression and must be surgically removed (1, 5). The present study reviews a case of intraspinal leiomyoma causing thoracic cord compression in a 19-year-old female. The clinical data were retrospectively evaluated, and a literature review of all other reported cases was performed.

## Case report

A 19-year-old female presented to the Beijing Tiantan Hospital (Capital Medical University, Beijing, China) with numbness of the right lower extremities and severe progressive weakness in the right leg that had been apparent for two months. There was no reported history of other diseases. Written informed consent was obtained from the patient’s family and study approval was obtained from the Institutional Review Board of Beijing Tiantan Hospital.

The neurological examination revealed a muscle power grade of 3/5 (as classified by the Medical Research Council grading system) (12) in the right leg and a grade of 4/5 in the left leg. Deep and superficial sensation below the T12 level was reduced. The muscle tone of the bilateral legs was increased and the deep tendon reflexes had hyper-excitability. Bilateral Babinski signs were present and sphincter function was normal.

**Figure 1:**
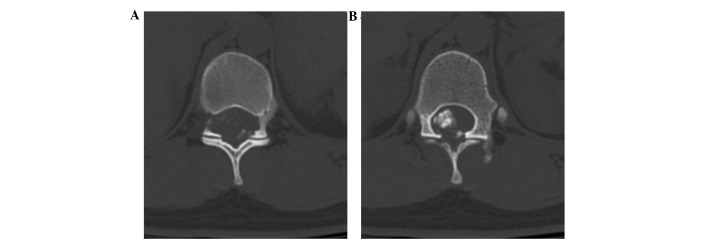
Axial computed tomography scan slice at thoracic level 11, showing extension of the neural foramina on the right, with (A) foraminal enlargement and (B) an isodense mass with calcification.

Abdominal computed tomography (CT) scans and an ultrasound of the uterus demonstrated normal results. CT of the spine disclosed an epidural mass with patchy calcification at the T11–12 level, causing compression and deviation of the spinal cord. An axial CT scan slice at the T11 level showed extension of the neural foramina on the right, with foraminal enlargement (Fig. 1).

**Figure 2:**
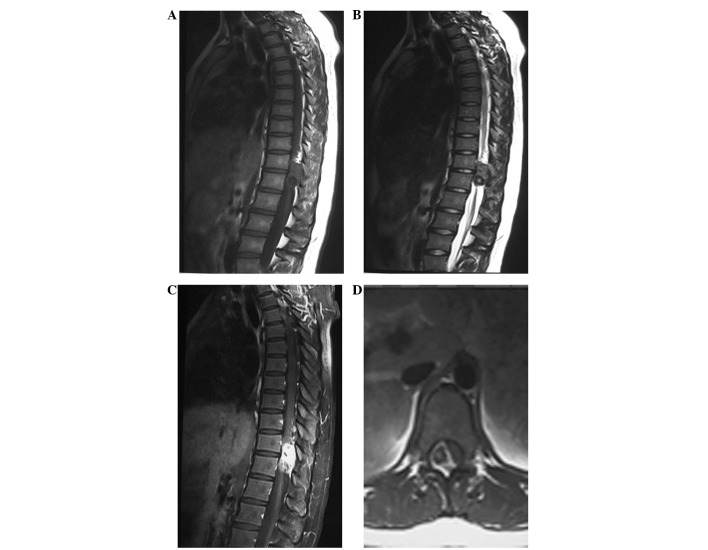
Pre-operative magnetic resonance imaging showing a mass with iso- to hypointensity on the (A) T1-weighted image and (B) mixed hypointensity on the T2-weighted image. (C) Heterogeneous enhancement on the T1-weighted image with gadolinium. (D) The spinal cord was severely compressed and displaced to the left.

On magnetic resonance imaging (MRI), the tumor was well-circumscribed, isointense on T1-weighted image and iso- to hypointensity on T2-weighted image (Fig. 2). Following gadolinium administration, the tumor showed heterogeneously marked enhancement. The spinal cord was severely compressed and displaced to the left, and cord edema was noted.

A T11–12 laminectomy was performed through the posterior approach. The tumor was located in the epidural space, with significant calcification and was firm and relatively avascular. Due to the well-demarcated dissection plane and mild adhesion to the dura, the tumor was completely removed.

**Figure 3:**
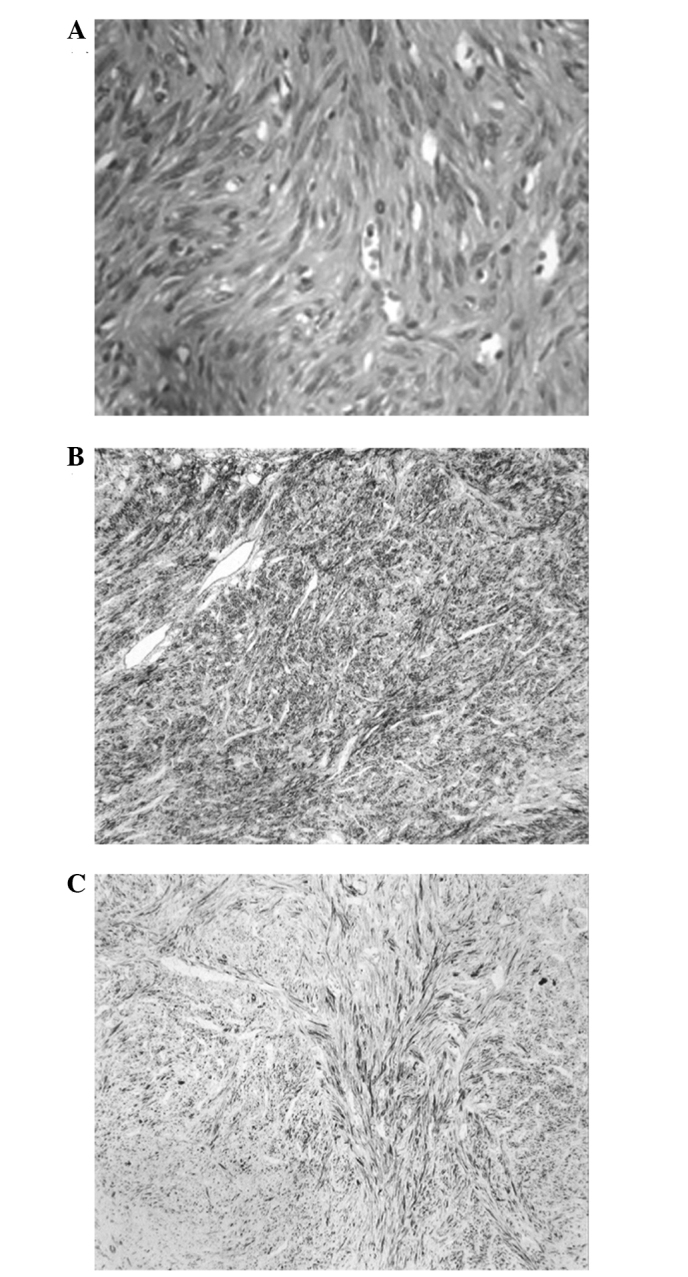
Photomicrographs of the surgical specimens illustrating (A) spindle-shaped cells arranged in fascicles with blunt-ended nuclei (hematoxylin and eosin staining; original magnification, ×400), (B) strong positivity for smooth muscle actin (immunohistochemical staining; original magnification, ×100) and (C) strong positivity for desmin (immunohistochemical staining; original magnification, ×100).

A histopathological examination revealed that the tumor was composed of intersecting fascicles of acidophilic spindle cells with blunt-ended nuclei, without significant cellular pleomorphism or mitotic activity (Fig. 3A). There were no visible signs of cellular atypia or necrosis. The immunohistochemical examinations revealed that the tumor cells were positive for smooth muscle actin (SMA) (Fig. 3B) and desmin (Fig. 3C). The Ki67 index was <2%, whereas staining for S-100 protein, estrogen and progesterone receptors was negative. Taken together, all of these findings were consistent with a diagnosis of leiomyoma.

As the nature of the tumor was benign, further treatment was not recommended. The numbness in the right lower limb of the patient was relieved following the surgical intervention and the patient was discharged 1 week later. The patient was able to walk unaided and no recurrence or regrowth of the tumor was observed on follow-up MRI after 25 months.

## Discussion

Without any histological malignant features, leiomyomas rarely metastasize. Leiomyoma metastasis to the spine is extremely rare. Only seven cases of benign metastasizing leiomyoma of the spine have been reported in the English literature (1–7) since the first case was described by Gatti *et al* in 1983 (3). Primary leiomyomas of the spine are even rarer, with only one case reported by Steel *et al* in 1993 (8). Table I summarizes the clinical features of previous cases of intraspinal leiomyoma, together with the present case.

The pathogenesis of intraspinal leiomyoma is poorly understood, but a few theories have been proposed. Steel *et al* (8) hypothesized that primary intraspinal leiomyoma may arise from blood vessel elements, either from within the spinal dura or from the epidural vessels. Other studies have suggested that the Epstein-Barr virus, the human immunodifficiency virus and immunosuppression may be cofactors in benign metastasizing leiomyoma (6,9–11).

In the present case, abdominal CT scans and an ultrasound of the uterus were performed to search for the origin of the metastasis. Although the results were normal, they were not sufficient to lead to the conclusion that the spine was the primary site of the tumor. It is well known that the spinal column and epidural region can harbor neoplasms of various pathological forms (8). The spinal tumor of the present case may have been metastatic and the primary tumor may have been clinically silent.

In the eight cases presented in the literature, the patients ranged in age between 9 and 56 years, and there was a female predominance (2 males and 6 females). All the lesions were located in the epidural space, with three involving the cervical segment, four involving the thoracic segment and one involving the sacral segment.

The signs and symptoms of spinal epidural leiomyomas are consistent with those of other epidural tumors. The clinical presentation of a spinal leiomyoma can include radicular pain, progressive or sudden weakness in the limbs, progressive spasticity and saddle anesthesia when the cauda equina is involved (3,6,7). The symptoms usually evolve over a period of weeks to years (1,6,8).

In the reported cases, the intraspinal leiomyomas showed isointensity on T1-weighted images and iso- to hypointensity on T2-weighted images (1,2,6). In certain cases, heterogeneous enhancement, with well-defined tumor margins, was detected following gadolinium administration. The imaging features identified in the present case were consistent with these previously reported characteristics.

Due to a lack of any particular features, definitive pre-operative diagnosis may be challenging based only on CT and MRI. Therefore, histopathological examination is required to differentiate leiomyomas from other common epidural lesions, including metastases, lymphoma and leiomyosarcoma.

The pathological features of the present case were typical of leiomyoma. The tumor was composed of interlacing fascicles of acidophilic cells, resembling a nerve-sheath tumor. Immunohistochemistry is often a necessary adjunct to make a differential diagnosis. Strong and diffuse immunostaining for SMA and desmin has been recognized as the most suitable and reliable diagnostic marker (4,5). No cellular atypia or necrosis was observed in the present case. The Ki67 index was <2%, therefore excluding the diagnosis of leiomyosarcoma. In the literature, immunostains are occasionally positive for oestrogen and progesterone receptors (1,5,13). However, the present case was immuno-negative for each of these receptors.

Due to the benign nature of the tumor, gross total resection is the optimal treatment for patients with symptomatic leiomyomas, in order to achieve a relatively good prognosis (14). The majority of spinal leiomyomas present with an intact capsule and are located in the epidural space (4,5,8). It is usually not difficult to achieve complete removal of the mass whilst avoiding damage to the adjacent nerves. In the reported cases, a gross total resection was achieved in seven patients and a subtotal resection in one case. The subtotal removal in this case was due to tight adherence of the tumor to the nerve roots.

Hormonal therapy is considered to be beneficial for preventing tumor recurrence, particularly when the histological examination is positive for the estrogen and progesterone receptors (1). The mechanism behind this has been indicated to be based on the feedback inhibition of estrogen secretion (4,6,15–17). In the literature, two cases underwent post-operative hormonal therapy, and one of these cases experienced tumor recurrence as the hormonal therapy was discontinued. The reason for the recurrence may be due to hormonal dependency or hormonal fluctuations (18,19).

The reported post-operative course ranged between 5 months and 13 years. Good outcomes were obtained following surgery in 4/5 cases with follow-up evaluations. Seven cases experienced gradual or complete functional improvement, and there was only one tumor recurrence. In the present case, the spinal cord was severely compressed, however, a good result was obtained following gross total resection.

In conclusion, although intraspinal leiomyoma is rare, it should be considered in the differential diagnosis of other common epidural lesions in females. Due to the potential for the patient to experience neurological recovery and be cured, clinicians and neurosurgeons should be aware of this pathology.

## Figures and Tables

**Table I:** Summary of previously reported spinal epidural leiomyoma cases.

First author, year (ref.)	Age, years/gender	Location	Clinical presentation	Duration of illness	Origin	MRI findings			Treatment	Estrogen and progesterone receptors	Follow-up

						T1WI	T2WI	+GA			
Gatti *et al,* 1983 (3)	56/F	C2-3	Neck pain	2 months	Uterine	NA	NA	NA	GTR	NA	18 months, CR, no rec
Steel *et al,* 1993 (8)	52/M	T3	Back pain	18 months	Primary spinal leiomyoma	NA	NA	NA	GTR	NA	NA
Hekster *et al,* 1994 (4)	43/F	C5-7	Left shoulder and hand pain	NA	Uterine	NA	NA	NA	STR + HT	NA	13 years, ICR, rec
Choi *et al,* 1997 (2)	9/M	T4	Paraparesis	7 months	Right foot and left axilla	Iso	Hypo	Heter	GTR	NA	NA
Joseph *et al*, 2003 (6)	38/F	C3-7	Progressive spasticity	12 months	Uterine	Hypo	Hypo	Heter	STR	Negative	5 months, CR, no rec
Alessi *et al,* 2003 (1)	42/F	S2	Saddle anesthesia/back pain	2 weeks	Uterine	Iso	Hypo	Heter	GTR + HT	Positive	12 months, CR, no rec
Vicente *et al,* 2005 (7)	36/F	T6	Paraparesis	NA	Uterine	NA	Iso to Hypo	NA	GTR	NA	NA
Jayakody *et al,* 2011 (5)	44/F	T5, T10	Thoracic pain	2 months	Uterine	NA	Iso to Hypo	NA	GTR	Positive	NA
Present study	18/F	T11-12	Right lower extremity numbness	2 months	Primary spinal leiomyoma	Iso	Iso to Hypo	Heter	GTR	Negative	23 months, CR, no rec

C, cervical; CR, complete remission; EPI, epidural; +GA, after gadolinium administration; F, female; GTR, gross total resection; Heter, heterogeneously enhancing; HT, hormonal therapy; Hypo, hypointensity; ICR, incomplete remission; Iso, isointensity; L, lumbar; M, male; MRI, magnetic resonance imaging; NA, not available; rec, recurrence, STR: subtotal resection; T, thoracic; WI, weighted image.
